# INTIMACY IN LATER LIFE: A POTENTIAL PATHWAY FOR IMPROVING WELL-BEING IN OLDER WOMEN

**DOI:** 10.1093/geroni/igz038.2255

**Published:** 2019-11-08

**Authors:** Renee J Flores

**Affiliations:** UT Houston McGovern Medical School, Houston, Texas, United States

## Abstract

Despite benefits to overall health and well-being, healthcare professionals’ knowledge and research is limited in regards to older women’s sexuality and intimacy desires. There are barriers that impede fulfilling these desires and lack of understanding hinders ways to address this issue, which negatively affects the well-being of older women. A sexuality and intimacy survey of 29 women between the ages of 60-86 revealed that the majority were having sex at least once a month and expressed the desire to increase the frequency of sexual encounters. These data suggests that later-life sexuality and intimacy encounters are important for some women. Recognizing these desires could prompt responses that could greatly influence the quality of life in older women. A broader public health discussion needs to occur in order to promote awareness and optimize overall well-being.

